# Intravesical colistin irrigation to treat multidrug-resistant *Acinetobacter baumannii* urinary tract infection: a case report

**DOI:** 10.1186/1752-1947-6-426

**Published:** 2012-12-28

**Authors:** Patricia Volkow-Fernández, Cecilia Franco Rodríguez, Patricia Cornejo-Juárez

**Affiliations:** 1 Department of Infectious Diseases, Instituto Nacional de Cancerología, Avenida San Fernando No. 22, Colonia Sección XVI, Tlalpan, 14080, México, DF, Mexico

## Abstract

**Introduction:**

*Acinetobacter baumannii* is a Gram-negative bacteria and a significant nosocomial pathogen in hospitals. Multidrug-resistant *A*. *baumannii* have emerged as a cause of nosocomial infections in critically ill patients. This microorganism has the ability to produce biofilms on different surfaces, which could explain their ability to persist in clinical environments and their role in device-related infections.

**Case presentation:**

We present the case of a 33-year-old Hispanic man with local invasive retroperitoneal leiomyosarcoma and right kidney exclusion along with femoral venous thrombosis, who was admitted for tumor resection. He had been receiving multiple nephrotoxic antibiotics for a long time (including tigecycline and colistimethate sodium) and had a persistent urinary infection related to multidrug-resistant *A. baumannii* (with susceptibility to colistimethate). Colistimethate was administered through a three-lumen urinary device for continuous urinary irrigation over seven days. Our patient did not refer to any adverse effects. A urine culture control taken at the end of the irrigation and another taken 10 days later were negative.

**Conclusion:**

Colistimethate sodium and other antimicrobials infused by urinary irrigation can be a good option in patients in whom parenteral administration could be toxic.

## Introduction

Multidrug-resistant (MDR) *Acinetobacter baumannii* has become an emergent pathogen for critically ill patients in intensive care units in the last decade, causing ventilator-associated pneumonia, bacteremia, urinary tract infections (UTIs) and surgical-site infections [1-3]. These microorganisms have the potential to produce biofilms on different surfaces, which could explain their ability to persist in clinical environments and their role in device-related infections [4]. The lack of new antibiotics with activity against these MDR strains has obliged infectious disease physicians to use older antibiotics, such as colistimethate [5,6]. Pharmacological knowledge of this old antibiotic remains limited because it was developed in the 1960s, prior to the establishment of current drug-development requirements, but it has known potential for nephro- and neurotoxicity, although inhaled and intraventricular administration has been described as useful [1]. Despite the frequency of UTIs caused by MDR *A*. *baumannii*, no information related to therapeutical intravesical colistimethate administration was found in the literature.

We present the case of a patient with a UTI caused by MDR *A*. *baumannii*, who was successfully treated with intravesical colistimethate.

## Case presentation

A 33-year-old Hispanic man with local invasive retroperitoneal leiomyosarcoma and right kidney exclusion along with femoral venous thrombosis was admitted for tumor resection. A vascular graft was performed during the surgery but our patient developed hypovolemic shock and acute renal failure in the immediate postoperative period and was acutely ill when admitted to the intensive care unit. He developed abdominal sepsis with isolation of MDR *A*. *baumannii*, vancomycin-resistant *Enterococcus faecium* and oxacillin-resistant *Staphylococcus aureus*. He received meropenem, vancomycin and colistimethate for 21 days. He showed clinical improvement but an increase in his serum creatinine level (1.9 mg/dL).

Eight days after the antimicrobials were withdrawn, *A*. *baumannii* was isolated from a bronchial aspirate. The patient had no fever and his chest X-ray was reported as normal. No antimicrobial therapy was started. Ten days later, he developed fever; MDR *A*. *baumannii* was isolated from his urine. Tigecycline was prescribed for 10 days and his symptoms resolved.

The patient was discharged after a 52-day hospital stay without an indwelling urethral catheter, 10 days after tigecycline withdrawal. Twelve days after his hospital discharge, he developed fever, dysuria and muddy urine. A microscopic analysis of his urine sediment showed pyuria and nitrates. MDR *A*. *baumannii* with susceptibility to colistimethate was isolated from his urine culture. A UTI was diagnosed according to the Centers for Disease Control and Prevention/National Healthcare Safety Network surveillance criteria [7]. All blood cultures were negative, and he did not have any surgical-site infections or decubitus ulcers.

We considered intravesical administration of colistimethate to spare his renal function because our patient had only one functional kidney and had received multiple nephrotoxic drugs. Colistimethate sodium (Colmesdant®, Stendhal Labs) 3.5 mg/kg was dissolved in a 500 cm^3^ saline solution for 12 h and administered through a triple intravesical catheter with continuous irrigation over seven days. The patient’s creatinine and ureic nitrogen at this point were 0.99 mg/dL and 8.1 mg/dL, respectively.

His urinary symptoms disappeared 48 h after starting the intravesical irrigation. He did not report any adverse effects while under treatment. Microscopic analysis of the urinary sediment was negative and urine cultures taken at the end of the irrigation, 10 days later and one month later were all negative.

## Conclusion

In our case, continuous intravesical administration of colistimethate over seven days proved useful to eradicate a MDR *A*. *baumannii* urinary infection. Colistimethate administered by urinary irrigation can be a good option in selected patients in whom parenteral administration could be toxic.

## Consent

Written informed consent was obtained from the patient for publication of this case report and the accompanying images. A copy of the written consent is available for review by the Editor-in-Chief of this journal.

## Competing interests

The authors declare that they have no competing interests.

## Authors’ contributions

PV-F contributed to the writing of the manuscript and revised it critically for important intellectual content. CF-R calculated, prepared and followed-up colistimethate sodium irrigation, and interpreted data. PC-J analyzed and interpreted the patient’s data and was a major contributor to the writing of the manuscript. All authors read and approved the final manuscript.

## Authors’ information

PV-F is an infectious diseases physician and Underdirector of the INCan Diagnostic and Therapeutic Services. CF-R is a chemist and pharmacist who prepares intravenous medications for hospitalized patients. PC-J is Chief of the INCan Infectious Diseases Department.
